# [Retracted] Resistant starch prevents tumorigenesis of dimethylhydrazine-induced colon tumors via regulation of an ER stress-mediated mitochondrial apoptosis pathway

**DOI:** 10.3892/ijmm.2026.5952

**Published:** 2026-08-10

**Authors:** Qiuyu Wang, Peng Wang, Zhigang Xiao

Int J Mol Med 41: 1887-1898, 2018; DOI: 10.3892/ijmm.2018.3423

Following the publication of this paper, it was drawn to the Editor's attention by a concerned reader that three of the data panels showing the results of immunofluorescence experiments in Fig. 4H on p. 1893 were strikingly similar to data in a paper that had previously been published in the journal *Nature Communications* that was written by different authors in different research institutes. Furthermore, an independent analysis of the data in this paper performed by the Editorial Office revealed that microscopic data in Fig. 4B and western blot data in Fig. 6D had also previously appeared in articles that were published by different authors at different research institutes.

Given that the abovementioned data had already been published in several different journals, the Editor of *International Journal of Molecular Medicine* has decided that this paper should be retracted from the Journal. The authors were asked for an explanation to account for these concerns, but the Editorial Office did not receive a reply. The Editor apologizes to the readership for any inconvenience caused.

